# Sustainable HR practices and Generation Z: role of career growth in commitment and turnover intention

**DOI:** 10.3389/fpsyg.2026.1819084

**Published:** 2026-05-18

**Authors:** Gönül Konakay, Sabiha Sevinç Altaş, Nurcan Günce, Ayşe Günsel, Meral Elçi

**Affiliations:** 1 Hereke Ömer Ismet Uzunyol Vocational School, Kocaeli University, Kocaeli, Türkiye; 2 Vocational School of Health Services, Sakarya University, Sakarya, Türkiye; 3 Business School, Kocaeli University, Kocaeli, Türkiye; 4 Department of Business Administration, Gebze Technical University, Kocaeli, Türkiye

**Keywords:** career growth, Generation Z, intention to quit, organizational commitment, sustainable HRM

## Abstract

**Introduction:**

This study examines how sustainability-oriented human resource management practices relate to perceived career growth, organizational commitment, and intention to quit among Generation Z employees.

**Methods:**

Drawing on a sample of 403 employed Gen Z respondents in Türkiye, we estimated a mediated model using PLS-SEM (SmartPLS 4) with 5,000 bootstrap resamples. Organizational commitment and intention to quit were modeled as distinct latent constructs, and the mediating role of career growth was explicitly tested.

**Results:**

Results indicate that SHRM is positively associated with perceived career growth, which in turn is linked to higher commitment and lower quitting intentions. The indirect (mediated) relationships were statistically significant, suggesting that career growth functions as a meaningful pathway through which SHRM is associated with attachment-related outcomes for this cohort.

**Discussion:**

Theoretically, the findings link sustainability-oriented HR bundles to a career-development mechanism that appears especially relevant for younger employees evaluating future opportunity within the organization. Practically, they point to codified career architectures, frequent progress reviews, and development-linked recognition as actionable levers to enhance engagement and retention. While the cross-sectional, single-country design limits causal and cross-context generalization, the results offer a useful basis for understanding how SHRM may be aligned with early-career employee expectations in evolving labor markets.

## Introduction

Sustainable Human Resource Management (SHRM) has gained prominence as organizations seek to align economic performance with employee wellbeing and longer-term social sustainability (Ehnert et al., 2016). Yet, an important question remains underdeveloped in this literature: through which psychological mechanism do sustainability-oriented HR systems become consequential for early-career employees' attachment to the organization? This question matters because formally well-designed HR architectures do not automatically produce favorable employee attitudes; their effects depend on how employees interpret whether the system offers a credible future inside the organization (Bowen and Ostroff, 2004; Nishii et al., 2008).

This issue is especially salient for employees commonly grouped under the label of Generation Z. Because birth-year boundaries vary across studies, we use Generation Z as a pragmatic cohort label rather than as a claim of strict generational homogeneity. Our focus is on early-career employees who entered the labor market under broadly comparable socio-economic conditions and who are therefore likely to be attentive to whether organizations offer development, continuity, and a believable path forward (Mannheim, 1952). For this group, the practical question is not merely whether supportive HR practices exist, but whether those practices can be experienced as a genuine basis for growth and continued membership.

We argue that perceived career growth is the primary mechanism through which SHRM becomes psychologically meaningful for such employees. Career growth captures employees' perceptions that their present work advances their longer-term career goals through skill development, advancement prospects, and material progression. This mechanism is theoretically important because it moves the discussion from broad claims about “good HR” to a more precise explanation of why sustainability-oriented HR systems should matter for commitment and intention to quit.

Our argument is grounded first in Social Cognitive Career Theory (SCCT), which explains how career-related expectations and goals are shaped by contextual supports and constraints (Lent et al., 1994). When employees encounter HR systems that provide developmental resources, regular feedback, participation opportunities, and a sustainable career climate, they are more likely to see career progress as feasible rather than uncertain. From this perspective, SHRM should strengthen perceived career growth because it makes advancement more attainable, interpretable, and worth investing in.

Our argument is grounded second in Psychological Contract Theory, which helps explain why career growth should connect SHRM to retention-relevant outcomes (Rousseau, 1995). When organizations are perceived as investing in employees in a consistent and future-oriented manner, employees are more likely to interpret this as a credible organizational commitment to their development. Such interpretations should foster stronger organizational commitment and reduce intention to quit. In this sense, career growth is not an ancillary benefit of SHRM; it is the channel through which sustainability-oriented HR systems are translated into attachment-related outcomes.

Accordingly, we specify a parsimonious model linking SHRM to organizational commitment and intention to quit through perceived career growth. This focus is deliberate. Rather than broadening the framework with additional unmeasured mechanisms, we center the analysis on the transmission path that is explicitly theorized and empirically tested in this study. Opportunity transparency may help interpret why some HR systems are experienced as more credible than others, but it is treated here only as a secondary interpretive lens, not as a focal construct.

Using survey data from 403 Generation Z employees in Türkiye, we test whether perceived career growth mediates the relationship between SHRM and two central attachment outcomes: organizational commitment and intention to quit. The study contributes in three ways. First, it sharpens the SHRM literature by identifying career growth as a concrete mechanism through which sustainability-oriented HR systems relate to retention-relevant attitudes. Second, it connects SHRM to SCCT and Psychological Contract Theory in a way that yields a tighter, more coherent explanation of why early-career employees stay committed or consider leaving. Third, it offers managerially actionable insight by showing that SHRM is most consequential when employees can connect developmental support to a believable future within the organization.

## Literature review

### Sustainable human resources practices and career growth among Generation Z employees

Sustainable Human Resource Management (HRM) practices have gained increasing prominence because they extend the focus of HR beyond short-term efficiency toward the long-term viability of employees, organizations, and broader stakeholder systems (Liang et al., 2022). In this sense, sustainable HRM is not merely a set of benevolent personnel practices; rather, it reflects an effort to reconcile economic objectives with social responsibility by creating employment systems that protect employee wellbeing while also supporting organizational performance (Stankeviciute and Savaneviciene, 2013; Mariappanadar and Kramar, 2014). The effectiveness of such practices, however, depends not only on their formal existence but also on whether employees perceive them as meaningful, coherent, and genuinely supportive of their future within the organization. Indeed, prior research suggests that stronger employee perceptions of sustainability-oriented HRM are associated with more favorable attitudinal and performance-related outcomes, including engagement and commitment (Jeronimo et al., 2020).

In line with this employee-centered perspective, we conceptualize sustainable HRM as an employee-perceived and integrated bundle of practices rather than as a set of isolated HR policies. Specifically, we draw on De Prins et al.'s (2020) Sustainable HRM scale, which captures three complementary dimensions: decent work, workplace democracy, and a sustainable career climate. These dimensions jointly reflect a sustainability-oriented HR architecture. Decent work emphasizes meaningful and person-job-aligned work design; workplace democracy emphasizes participation, voice, and inclusive decision processes; and a sustainable career climate emphasizes employability support through development opportunities, regular feedback, and career-relevant learning. Considered together, these dimensions signal whether the organization invests in employees not only as current performers but also as long-term contributors whose development matters over time.

This developmental signal is especially important when examining career growth. Career growth refers to employees' perceptions that they are able to build skills, assume broader responsibilities, and move toward valued career goals over time (Baluch and Ridder, 2021). Although contemporary careers are increasingly shaped by mobility across roles and organizations rather than by purely linear advancement within a single employer, employees still assess current workplaces in terms of whether they provide the learning conditions, feedback structures, and developmental support necessary for future progress (McDonald and Hite, 2014). In this respect, career growth remains a central indicator of whether an employment system is experienced as developmentally enabling rather than merely administratively efficient. It also carries intrinsic value, because growth-related experiences contribute to employees' sense of purpose, capability development, and alignment between work and broader career aspirations (Dik et al., 2015).

For Generation Z employees, these issues are likely to be particularly salient. As early-career employees, Generation Z workers are especially attentive to cues about employability, development opportunities, and future progression when evaluating the quality of the employment relationship. Accordingly, sustainable HRM practices should be more likely to foster career growth when employees perceive them as a coherent and future-oriented HR architecture. When employees experience meaningful work, participation opportunities, and a supportive career climate together, they are more likely to interpret the organization as one that offers a credible basis for learning, advancement, and longer-term development. Under such conditions, sustainable HRM practices should strengthen employees' perceptions that effort, learning, and participation can translate into future progress. Therefore, employee-perceived sustainable HRM practices are expected to be positively associated with career growth among Generation Z employees.

H1: There is a positive relationship between sustainable HRM practices and career growth among Generation Z employees.

### Sustainable human resource practices and organizational commitment among Generation Z employees

Organizational commitment refers to the psychological bond that connects employees to their organization and shapes a range of important work outcomes, including performance, retention, and extra-role behaviors (Meyer and Allen, 1991). As one of the most widely studied constructs in organizational behavior, organizational commitment captures the extent to which employees identify with organizational goals and are willing to maintain their membership in the organization (Meyer and Allen, 1991). The concept is commonly examined through three dimensions—affective, continuance, and normative commitment—each reflecting a distinct basis for attachment (Meyer and Allen, 1991). In the context of this study, organizational commitment is especially relevant because it reflects whether Generation Z employees develop a meaningful and enduring attachment to organizations that seek to retain them in an increasingly competitive labor market.

As Generation Z becomes more visible in the workforce, organizations are under growing pressure to design HR systems that do more than attract young talent; they must also sustain employees' attachment over time. Generation Z is commonly discussed as a cohort entering the workforce from the late 2010s onward, although its birth-year boundaries vary across studies, generally spanning from the mid-1990s to the early 2010s (Dimock, 2019; McCrindle, 2019, 2021; Seemiller and Grace, 2016; Bencsik et al., 2016). Prior research suggests that members of this cohort are particularly attentive to developmental opportunities, meaningful work environments, and organizational values that extend beyond short-term economic exchange. For this reason, commitment among Generation Z employees is unlikely to be strengthened by isolated HR practices alone; rather, it is more likely to emerge when employees perceive HRM as a coherent, long-term, and employee-supportive system.

Research has consistently shown that HR practices such as training and development, compensation, information sharing, and effective communication are positively associated with stronger employee attachment to the organization (Gellatly et al., 2009; Wright and Kehoe, 2008). High-commitment HR practices are often interpreted by employees as signals that the organization values their contribution and intends to invest in their future, which in turn enhances organizational commitment (Akingbola, 2013). In a similar vein, Pratama et al. (2023) argue that well-designed HR practices foster employee engagement, which subsequently supports stronger commitment. Sustainable HRM extends this logic by emphasizing not only organizational performance, but also employee wellbeing, voice, and long-term development (Leslie et al., 2021; Meret et al., 2018). Such practices are therefore especially relevant for understanding commitment among younger employees who may be more likely to evaluate employers in terms of developmental support, organizational values, and future prospects.

This argument is particularly salient for Generation Z. Although this cohort is often described as ambitious and success-oriented, recent studies also show that its members are attentive to health and safety, working hours, interpersonal climate, and the broader quality of the work environment (Guptill et al., 2023). Other studies indicate that Generation Z employees are more likely to value friendly workplaces, advanced technologies, opportunities for professional development, and involvement in socially responsible initiatives (Benítez-Márquez et al., 2022; Meret et al., 2018). When HR practices are aligned with these expectations, employees are more likely to perceive the organization as supportive, credible, and worth committing to. In this sense, sustainable HRM may strengthen organizational commitment by signaling that the organization is concerned not only with immediate performance, but also with employees' longer-term wellbeing, participation, and development.

Taken together, sustainable HRM can be expected to foster stronger commitment among Generation Z employees because it communicates a more enduring and reciprocal employment relationship. Rather than treating employees as short-term inputs, sustainable HRM frames them as long-term organizational contributors whose wellbeing, voice, and growth matter. Such perceptions are likely to deepen employees' psychological attachment to the organization and strengthen their willingness to remain part of it. Accordingly:

H2: There is a positive relationship between sustainable HRM practices and organizational commitment among Generation Z employees.

### Sustainable human resource practices and intention to quit among Generation Z employees

Intention to quit, often referred to as turnover intention, is a central construct in organizational behavior and human resource management because it captures employees' conscious consideration of leaving their current organization and serves as a proximal antecedent of actual turnover (Chang et al., 2017). Although intention to quit does not necessarily translate into immediate departure, it reflects an important cognitive evaluation of whether continued organizational membership remains desirable (Wong and Wong, 2017). Because such intentions are shaped by workplace experiences and organizational signals, they provide an especially useful indicator for understanding retention-related outcomes in contemporary organizations (Cohen et al., 2016; Coetzee et al., 2016). For organizations seeking to retain younger employees, examining the factors that reduce intention to quit is therefore both theoretically and practically important.

This issue is particularly salient for Generation Z employees, whose workplace decisions are often shaped by their evaluation of developmental opportunities, work–life balance, social responsibility, and person-environment fit (Safian et al., 2021; Schroth, 2019; Ayoobzadeh et al., 2024). Prior research suggests that when work environments are perceived as misaligned with these expectations, younger employees may become more willing to disengage and consider leaving. Conversely, when organizations offer meaningful work, developmental support, and a socially embedded work context, employees are more likely to develop stronger attachment and lower quitting intentions (Chillakuri, 2020). In this sense, turnover intention among Generation Z employees is likely to be shaped not only by immediate job conditions, but also by whether organizational practices signal a viable and supportive future within the organization.

A substantial body of research has linked HR systems to lower intention to quit. High-performance and supportive HR practices—such as clear communication, recognition, and growth opportunities—have been shown to reduce turnover risk by creating a more attractive and supportive employment context (Kehoe and Wright, 2013). Similarly, career-advancement support and work-life balance practices can enhance job satisfaction and reduce employees' desire to leave, particularly when HR strategies are aligned with employees' aspirations and wellbeing (Ayoobzadeh et al., 2024; Mahardika et al., 2023). These insights are especially relevant for Generation Z, whose retention appears to depend heavily on whether organizations provide not only employment, but also developmental and value-based reasons to stay.

Within this context, sustainable human resource management (SHRM) offers a particularly relevant framework for understanding lower quitting intentions among Generation Z employees. By emphasizing long-term employee wellbeing, development, participation, and responsible people management, SHRM may reduce intention to quit in several mutually reinforcing ways. First, sustainable HRM can strengthen employees' perceptions that the organization is invested in their future through learning opportunities, career support, and fair developmental practices. Second, it can enhance the quality of the work environment by supporting healthier and more balanced working conditions. Third, it can increase value congruence by signaling that the organization is concerned not only with short-term performance, but also with employee dignity, voice, and sustainability-oriented values (Leslie et al., 2021; Meret et al., 2018; Aggarwal et al., 2022). For Generation Z employees, who are often attentive to both personal development and broader organizational values, such signals are likely to reduce withdrawal cognitions and make continued membership more attractive.

Taken together, sustainable HRM practices can be expected to lower intention to quit among Generation Z employees because they create a more supportive, developmental, and future-oriented employment relationship. When employees perceive that the organization offers conditions under which they can grow, contribute, and maintain wellbeing, they are less likely to consider leaving. Accordingly:

H3: There is a negative relationship between sustainable HRM practices and intention to quit among Generation Z employees.

### The mediator role of career growth

Career growth refers to employees' perceptions that their current organization provides meaningful opportunities for professional advancement, skill development, and longer-term career progression (Vande Griek et al., 2020). As a developmental work outcome, career growth is especially important because it connects employees' personal aspirations with organizational opportunities, thereby shaping how they evaluate their future within the organization. Prior research suggests that when employees perceive stronger career growth prospects, they are more likely to experience higher job satisfaction, stronger attachment, and lower withdrawal tendencies (Spagnoli, 2020; Weng and McElroy, 2012). For Generation Z employees, career growth may be particularly salient, as this cohort is often described as highly attentive to learning, advancement, meaningful work, and self-development in the early stages of working life (Dimock, 2019; Xueyun et al., 2023). In this sense, career growth is not merely an additional job benefit, but a central lens through which younger employees evaluate whether the organization offers a viable future.

A positive relationship between career growth and organizational commitment is theoretically plausible because perceptions of growth signal that the organization provides room for development, recognizes employee potential, and supports long-term progression. Employees who perceive robust advancement opportunities are more likely to develop stronger emotional attachment and loyalty toward the organization, as such opportunities reinforce the sense that continued membership is both meaningful and worthwhile (Weng and McElroy, 2012). Weer and Greenhaus (2020) further argue that career growth perceptions are especially influential for individuals who are highly invested in their career paths, creating a mutually reinforcing dynamic in which growth enhances commitment and commitment encourages continued development. This logic is particularly relevant for Generation Z employees, for whom career development and professional advancement are often seen as indispensable elements of a satisfying employment relationship (Dimock, 2019; Xueyun et al., 2023). When organizations provide visible and credible opportunities for development, younger employees are more likely to interpret these experiences as evidence that the organization is invested in their future, thereby strengthening organizational commitment (Ertas, 2015). Accordingly, we expect a positive association between career growth and organizational commitment among Generation Z employees.

H4: There is a positive relationship between career growth and organizational commitment among Generation Z employees.

Career growth is also likely to reduce employees' intention to quit. Research consistently shows that employees who perceive clearer opportunities for advancement and development are less likely to consider leaving their organizations (Huo, 2021; Wang et al., 2024). When organizations actively support career progression, employees are more likely to view staying as beneficial for both their professional development and longer-term career goals. In this way, career growth can operate as a powerful retention mechanism by making continued organizational membership more attractive than exit (Valdez and Limos-Galay, 2023). Prior work likewise indicates that well-designed career development programs contribute to lower turnover by increasing employees' willingness to remain with the organization and by reinforcing their sense of future fit within it (Ng et al., 2024; Balushi et al., 2022).

This relationship may be even more pronounced for Generation Z employees. Because this cohort tends to place strong emphasis on learning opportunities, adaptability, meaningful work, and future advancement, the absence of career growth opportunities may quickly trigger doubts about staying, whereas the presence of such opportunities may strengthen retention (Dimock, 2019). Marozva et al. (2024) similarly highlights career growth as an important retention factor for Generation Z, showing that organizations with structured and well-defined progression paths tend to experience lower turnover among younger employees. For this cohort, career growth is therefore not simply a desirable feature of work; it is a concrete indicator that the organization can support their future aspirations. Accordingly, higher perceptions of career growth should be associated with lower intention to quit among Generation Z employees.

H5: There is a negative relationship between career growth and intention to quit among Generation Z employees.

Taken together, the above arguments suggest that career growth may serve as an important explanatory mechanism linking sustainable HRM practices to organizational commitment among Generation Z employees. Sustainable HRM is likely to enhance employees' perceptions that the organization offers meaningful developmental opportunities, longer-term progression, and support for future advancement. When such perceptions are strengthened, employees are more likely to feel valued, supported, and psychologically connected to the organization, which in turn reinforces their organizational commitment. In this sense, career growth provides a theoretically meaningful pathway through which sustainable HRM may translate into stronger attachment among younger employees. Accordingly:

H6: Career growth mediates the relationship between sustainable HRM practices and organizational commitment among Generation Z employees.

Career growth may also explain how sustainable HRM practices reduce intention to quit among Generation Z employees. If sustainable HRM fosters employees' perceptions that they can develop, advance, and build a future within the organization, employees are less likely to evaluate exit as a desirable option. For younger employees, who often place strong emphasis on development and future opportunity, perceived career growth may be especially important in transforming supportive HR practices into lower withdrawal cognitions. Accordingly, career growth is expected to function as a key mediating mechanism through which sustainable HRM decreases intention to quit among Generation Z employees.

H7: Career growth mediates the relationship between sustainable HRM practices and the intention to quit among Generation Z employees.

## Methodology

### Measures

To test the hypotheses, we used multi-item scales adapted from established studies. Each construct was measured on a five-point Likert scale ranging from 1 (“strongly disagree”) to 5 (“strongly agree”). Except for the turnover-intention scale, which had an established Turkish validation, all instruments were translated into Turkish and then back-translated into English by bilingual experts prior to data collection. Any discrepancies between the original and back-translated versions were reviewed and resolved to ensure conceptual equivalence, clarity, and linguistic accuracy. In addition, the Turkish versions were checked for item clarity during the pretest stage. The full list of items and their standardized loadings is reported in the Appendix.

Sustainable HRM practices were measured using the scale developed by De Prins et al. (2020). The instrument includes 11 items and captures three first-order dimensions: decent work (four items), workplace democracy (four items), and sustainable career climate (three items). Example items include “Jobs are a reflection of what employees are good at and like to do in our organization,” “The organization has no unnecessary hierarchical levels,” and “Employees receive regular feedback on their performance and results.” Consistent with our conceptualization, sustainable HRM was modeled as a reflective–reflective higher-order construct.

Career growth was measured using the Career Growth Scale developed by Weng et al. (2010). The scale comprises 15 items across four first-order dimensions: career goal progress (four items), professional ability development (four items), promotion speed (four items), and remuneration growth (three items). Illustrative items include “My present job moves me closer to my career goals,” “My present job enables me to continuously improve my professional capabilities,” “My promotion speed in the present organization is fast,” and “My salary is growing quickly in my present organization.” In line with our conceptual model, career growth was specified as a reflective–reflective higher-order construct.

Organizational commitment was assessed using the Organizational Commitment Scale developed by Allen and Meyer (1996). The original instrument comprises 18 items representing three dimensions: affective commitment, continuance commitment, and normative commitment, each measured with six items. Example items include “I would be very happy to spend the rest of my career in this organization,” “I believe I have too few options to consider leaving this organization,” and “This organization deserves my loyalty.” Organizational commitment was likewise modeled as a reflective–reflective higher-order construct. As reported in the **Appendix**, a small number of items were dropped during measurement model refinement based on standard psychometric criteria.

Intention to quit was measured using the scale originally developed by Bothma and Roodt (2013) and validated for Turkish use by Erkmen and Kahraman (2021). The scale was administered with eight reverse-worded items so that higher scores indicate lower intention to quit. Example items include “I look forward to another day at work,” “I never consider leaving my job,” and “After completing the task I am currently responsible for, I would prefer to stay with this company rather than leave.” Accordingly, positive coefficients involving this construct should be interpreted as reductions in intention to quit. This reverse-coded administration was used ex ante as a procedural step to reduce the underreporting of a sensitive withdrawal-related attitude and to lessen acquiescence tendencies.

The conceptual and methodological rationale for the higher-order specification is presented in Section 3.5.

### Sample and procedure

The study focused on Generation Z employees working in the Turkish private sector. To access a heterogeneous pool of early-career employees from one of the country's most economically active regions, we used convenience sampling in the Marmara Region of Türkiye (cf. Turkish Statistical Institute, 2023). Data were collected face-to-face between July and August 2024 in workplace settings and union-affiliated venues. Trade-union member registries were used solely to facilitate initial contact and scheduling; they were not used as analytical data sources. Participation was voluntary and anonymous, and respondents were informed that their answers would be used only for research purposes. Questionnaires were completed individually during the face-to-face sessions, and participants were informed that there were no right or wrong answers and that they could discontinue participation at any time without any consequence. No identifying information was collected on the questionnaire forms, and responses were not shared with employers, supervisors, or union representatives.

Eligibility criteria required participants to be currently employed and at least 18 years old. Individuals who were in full-time education without concurrent employment were excluded. Of the 600 employees approached, 442 agreed to participate (73.7%). Of these, 412 completed the questionnaire (93.2% of those who consented). Nine cases were removed because of incomplete responses, resulting in a final analytic sample of 403 employees, corresponding to an effective response rate of 67.2% of those initially contacted.

In line with commonly used operational definitions, we defined Generation Z as individuals born between 1995 and 2010 (Seemiller and Grace, 2016; Bencsik et al., 2016). In the present dataset, however, this cohort is represented primarily by its younger segment, as respondents' self-reported ages were concentrated between 18 and 24 years at the time of data collection (July–August 2024), corresponding approximately to birth years 2000–2006. We therefore use “Generation Z” as a pragmatic cohort label for this sample rather than as a claim of complete within-cohort homogeneity, and we acknowledge the possibility of meaningful variation within the cohort itself (e.g., younger vs. older Gen Z) (Mannheim, 1952; Costanza et al., 2012; Rudolph et al., 2021). Because the sample is concentrated in a relatively narrow age band, the study should be interpreted as examining variation within an early-career Generation Z group rather than estimating independent age effects across broader workforce cohorts. At the same time, organizational tenure still varies within this bounded life stage, indicating that respondents differ in their degree of workplace experience despite belonging to a relatively homogeneous age segment.

Although respondents were drawn from multiple industries, sector was not recorded at the individual level in this wave. We therefore avoid sector-specific inferences and treat this omission as a limitation on the generalizability of the findings.

Table 1 presents the demographic characteristics of the respondents, including gender, age, education level, position level, and organizational tenure.

**Table 1:** Demographics of the sample.

Variable	Category	*N*	(%)
Gender	Female	193	47.9
	Male	210	52.1
Age	18	41	10.2
	19	39	9.7
	20	57	14.1
	21	51	12.7
	22	59	14.6
	23	62	15.4
	24	94	23.3
Education level	High school	202	50.1
	Bachelor's degree	181	44.9
	Master's/doctorate	20	5.0
Position level	Employee	379	94.0
	Manager	24	6.0
Total tenure	Less than 1 year	103	25.6
	1–2 years	125	31.0
	3–5 years	88	21.8
	6 years or more	87	21.6

### Analysis

We tested the proposed model using partial least squares structural equation modeling (PLS-SEM) in SmartPLS 4.0 with bootstrapping (5,000 resamples). PLS-SEM is well-suited for prediction-oriented modeling and for estimating hierarchical latent variable models. Accordingly, SHRM was modeled as a reflective–reflective second-order construct and estimated using the repeated indicators approach in SmartPLS 4.0. We report measurement model quality (reliability, convergent, and discriminant validity), structural model results (standardized path coefficients with *t*-values, *p*-values, and confidence intervals), effect sizes, predictive relevance, and out-of-sample predictive performance. We followed established PLS-SEM procedures for model estimation and inference (Chin, 1998; Hair et al., 2013). Consistent with our theoretically narrow specification (SHRM → perceived career growth → outcomes), we did not include demographic covariates in the focal model; avoiding routine controls reduces the risk of over-control/post-treatment bias and aligns with recommendations that controls should be included only with strong theoretical justification (Bernerth and Aguinis, 2016; Spector and Brannick, 2011).

PLS-SEM was selected for three reasons. First, it is well-suited to models estimated with modest sample sizes and to applications that mix discrete and continuous indicators while modeling multiple endogenous constructs under explicit measurement error (Hair et al., 2021). Second, PLS-SEM imposes comparatively mild distributional assumptions on the data-generating process, which is advantageous when the distribution of latent variables is uncertain or non-normal (Hair et al., 2017). Third, relative to covariance-based SEM, PLS-SEM prioritizes prediction and can yield parameter estimates that align more closely with observed data patterns in complex models (Cha, 1994).

Following current recommendations, model assessment proceeded in two stages. We first evaluated the measurement model (indicator loadings, Cronbach's α, composite reliability, AVE; discriminant validity via Fornell–Larcker and HTMT; multicollinearity via inner VIFs), and then assessed the structural model using bootstrapped inference (5,000 resamples), reporting path coefficients (β), *t*-statistics, *p*-values, and confidence intervals; overall fit was summarized with SRMR (Hair et al., 2013).

### Measurement validation

This study used reflective measurement models for all constructs. To evaluate the psychometric properties of the measures, we assessed the measurement model prior to interpreting the structural relationships. Internal consistency reliability was evaluated using composite reliability (CR), Cronbach's alpha, and McDonald's omega whereas convergent validity was assessed using indicator (outer) loadings and average variance extracted (AVE). During item purification, one indicator from continuance commitment and two indicators from normative commitment were removed because they exhibited insufficient loadings and/or reduced AVE below the recommended minimum (0.50). After these adjustments, all constructs demonstrated satisfactory reliability and convergent validity: CR values exceeded 0.70, Cronbach's alpha values exceeded 0.70, and AVE values were above (or very close to) 0.50 (Table 2). In addition to Cronbach's alpha and composite reliability, McDonald's omega values were calculated for the retained reflective first-order dimensions. All omega coefficients were above 0.70, indicating acceptable internal consistency, and most were substantially higher, suggesting strong reliability at the subdimension level (Hair et al., 2020). Finally, the retained indicators showed adequate standardized outer loadings on their intended constructs (all > 0.60). A content-validity check confirmed that removing these indicators did not narrow the conceptual domain of the constructs, as the retained items continued to cover the intended facets of each scale.

**Table 2:** Descriptive statistics, reliability, and Fornell–Larcker criterion.

Construct	Mean	SD	CR	Alpha	AVE	Omega	1	2	3	4	5	6	7	8	9	10	11
Affective commitment	2.399	1.486	0.921	0.938	0.718	0.986	**0.847**										
Career goal progress	2.329	1.608	0.897	0.929	0.765	0.982	0.617	**0.874**									
Continuance commitment	2.318	1.262	0.799	0.869	0.625	0.938	0.762	0.616	**0.791**								
Decent work	2.468	1.512	0.839	0.893	0.676	0.970	0.555	0.555	0.572	**0.822**							
Turnover intention (reverse-coded; higher = lower turnover intention)	3.806	1.271	0.896	0.916	0.578	0.964	0.740	0.615	0.779	0.613	**0.760**						
Normative commitment	2.395	1.067	0.859	0.899	0.640	0.967	0.754	0.586	0.749	0.569	0.739	**0.800**					
Professional ability development	2.525	1.638	0.900	0.930	0.770	0.986	0.612	0.661	0.549	0.519	0.576	0.508	**0.877**				
Promotion speed	2.129	1.293	0.830	0.887	0.662	0.967	0.613	0.582	0.640	0.568	0.623	0.580	0.497	**0.814**			
Remuneration growth	2.148	1.477	0.866	0.918	0.790	0.981	0.629	0.572	0.692	0.585	0.660	0.626	0.538	0.684	**0.889**		
Sustainable career climate	2.592	1.687	0.852	0.910	0.772	0.968	0.606	0.621	0.572	0.583	0.598	0.517	0.635	0.526	0.538	**0.879**	
Workplace democracy	2.477	1.522	0.852	0.900	0.692	0.968	0.622	0.521	0.671	0.698	0.678	0.627	0.546	0.595	0.644	0.591	**0.832**

Diagonal (bold) values are the square roots of AVE; off-diagonal values are correlations.

Turnover intention is reverse-coded such that higher values indicate lower intention to quit.

To contextualize the measurement properties, Table 2 reports means (*M*) and standard deviations (SD) alongside the correlation matrix (secondary firm-performance data excluded). Construct means were modest on average—ranging from 2.13 (Promotion speed) to 3.81 (intention to quit)—with dispersion between SD = 1.07 and SD = 1.27, indicating substantial between-respondent heterogeneity across focal domains. Internal consistency was satisfactory: for all constructs, composite reliability (CR) and Cronbach's α exceeded conventional thresholds (CR, α ≥ 0.80), and av-erage variance extracted (AVE) values were ≥0.50 (see Table 2), supporting convergent validity.

Discriminant validity was evaluated using the Fornell–Larcker criterion (Fornell and Larcker, 1981). As shown in Table 2, the square root of AVE (diagonals) exceeds all inter-construct correlations in the corresponding rows/columns, indicating that each scale shares more variance with its intended construct than with any other (Hair et al., 2021). This pattern confirms satisfactory discriminant validity under the Fornell–Larcker test.

As a robustness check, we further examined the Heterotrait–Monotrait (HTMT) ratios of correlations (Henseler et al., 2015). Table 3 presents HTMT values for all construct pairs. All ratios were ≤ 0.886, remaining below the commonly used 0.90 benchmark for conceptually related reflective constructs, thus supporting discriminant validity. At the same time, one theoretically proximal pair—Affective commitment–Continuance commitment (HTMT = 0.886)—exceeds the more conservative 0.85 threshold, and several pairs approach it (e.g., Affective–Normative = 0.844; Remuneration growth–Continuance = 0.828; Workplace democracy–Decent work = 0.822; Intention to quit–Continuance = 0.820). Collectively, these results support discriminant validity under the 0.90 rule-of-thumb, while also reflecting substantive relatedness among commitment facets and employment-condition perceptions—relationships that are theoretically plausible in this context.

**Table 3:** Discriminant validity (HTMT).

Construct	1	2	3	4	5	6	7	8	9	10	11
Affective commitment											
Career goal progress	0.676										
Continuance commitment	0.886	0.726									
Decent work	0.628	0.638	0.698								
Turnover intention (reverse-coded)	0.813	0.684	0.820	0.703							
Normative commitment	0.844	0.666	0.713	0.661	0.837						
Professional ability development	0.669	0.732	0.645	0.595	0.636	0.575					
Promotion speed	0.697	0.672	0.784	0.678	0.718	0.681	0.570				
Remuneration growth	0.702	0.648	0.828	0.684	0.745	0.721	0.605	0.803			
Sustainable career climate	0.683	0.711	0.694	0.687	0.679	0.600	0.721	0.623	0.622		
Workplace democracy	0.703	0.594	0.813	0.822	0.771	0.726	0.622	0.706	0.748	0.691	

HTMT values below 0.90 (and ideally below 0.85) indicate discriminant validity.

### Higher-order construct specification

Sustainable HRM practices, career growth, and organizational commitment were modeled as reflective–reflective higher-order constructs. This specification was adopted because, in each case, the lower-order dimensions were conceptualized as related manifestations of a broader latent construct rather than as independent components requiring separate structural roles. In other words, the dimensions were expected to covary because they reflect a common underlying phenomenon experienced by employees at a more general level.

This logic is particularly important for sustainable HRM practices. In the present study, sustainable HRM was not treated as a collection of isolated HR practices, but as an integrated sustainability-oriented HR architecture. Decent work, workplace democracy, and sustainable career climate capture distinguishable yet theoretically connected aspects of how employees experience this broader HR system. Modeling these dimensions as separate standalone predictors would therefore fragment a construct that is conceptually more coherent at the system level.

A similar rationale applies to career growth and organizational commitment. Career growth is reflected in employees' perceptions of career goal progress, professional ability development, promotion speed, and remuneration growth, which together indicate whether the organization is experienced as offering a meaningful trajectory of advancement. Likewise, organizational commitment is conventionally represented by affective, continuance, and normative dimensions, which capture different but related forms of attachment to the organization. In both cases, the lower-order dimensions are theoretically linked and are more appropriately represented as manifestations of broader constructs.

**Figure 1:**
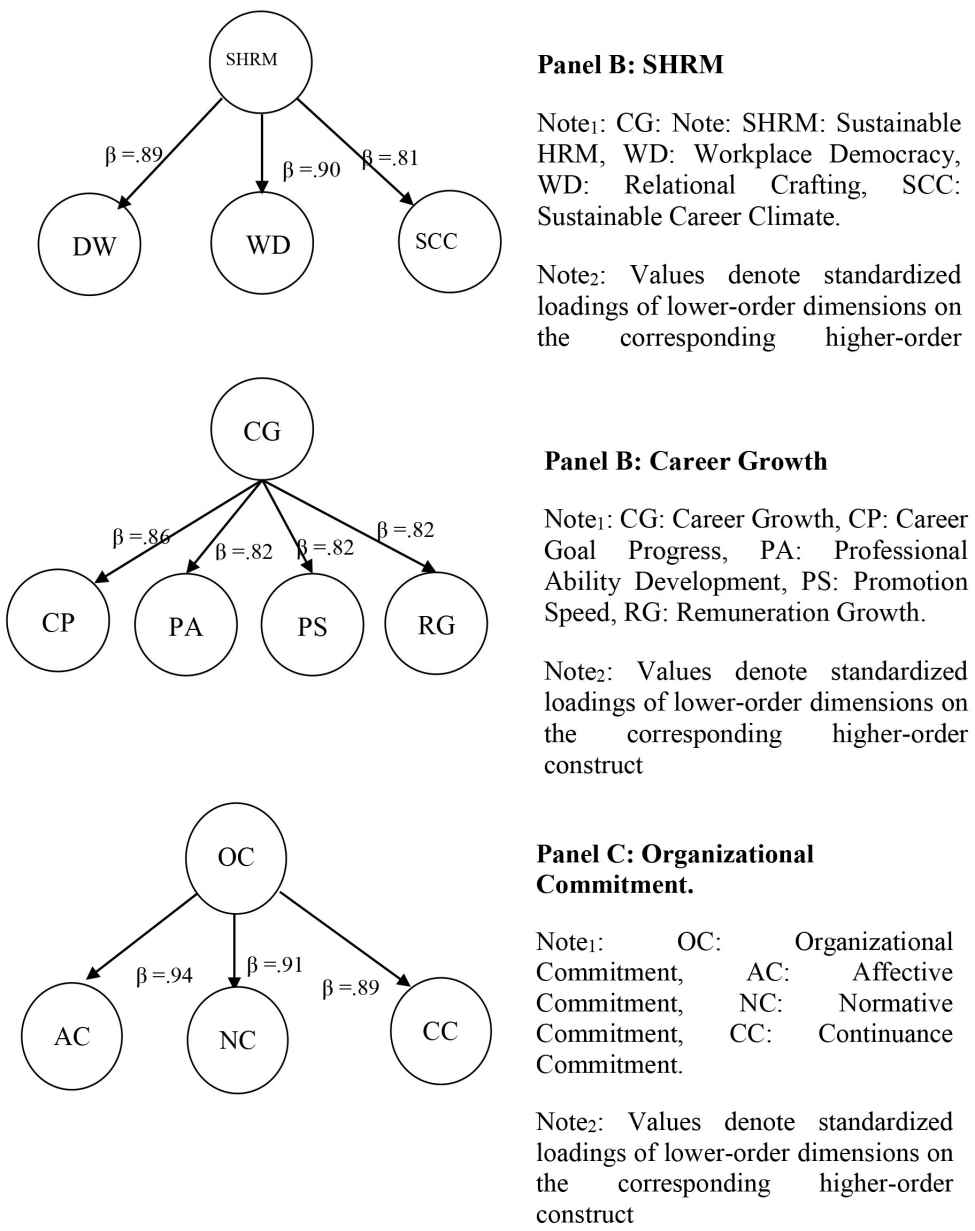
Higher-order construct specifications. **(A)** SHRM. CG, Career Growth; SHRM, Sustainable HRM; WD, Workplace Democracy; WD, Relational Crafting; SCC, Sustainable Career Climate. Values denote standardized loadings of lower-order dimensions on the corresponding higher-order construct. (**B)** Career growth. CG, Career Growth; CP, Career Goal Progress; PA, Professional Ability Development; PS, Promotion Speed; RG, Remuneration Growth. Values denote standardized loadings of lower-order dimensions on the corresponding higher-order construct. **(C)** Organizational commitment. OC, Organizational Commitment; AC, Affective Commitment; NC, Normative Commitment; CC, Continuance Commitment. Values denote standardized loadings of lower-order dimensions on the corresponding higher-order construct.

Methodologically, these higher-order constructs were estimated in SmartPLS 4.0 using the repeated indicators approach, whereby the indicators of the lower-order dimensions were assigned to the corresponding higher-order construct in order to estimate the hierarchical component model. This approach is consistent with PLS-SEM guidance when lower-order dimensions are expected to covary and collectively represent an overarching construct. Figure 1 summarizes these higher-order measurement results.

As shown in Panel A, sustainable HRM practices are represented by three first-order dimensions—decent work, workplace democracy, and sustainable career climate—and the standardized loadings of these dimensions on the higher-order construct exceed the commonly used 0.60 threshold. Panel B reports the higher-order results for career growth, which is represented by four dimensions—career goal progress, professional ability development, promotion speed, and remuneration growth—with all standardized loadings exceeding 0.60. Finally, Panel C shows that organizational commitment is represented by affective, normative, and continuance commitment, again with standardized loadings above 0.60. Collectively, these results support the adequacy of the specified higher-order constructs in the measurement model.

Harman's single-factor test showed that the first unrotated factor explained 39.17% of the variance (<50%), suggesting CMV is unlikely to be a dominant concern. As a complementary check, Kock's full-collinearity test indicated VIF values between 1.11 and 2.64 (<3.3), further reducing CMV concerns. Inner VIF values for the predictor constructs were also below conservative cutoffs (e.g., <3.3), indicating no problematic multicollinearity that could bias the path estimates.

### Hypothesis testing

**Figure 2:**
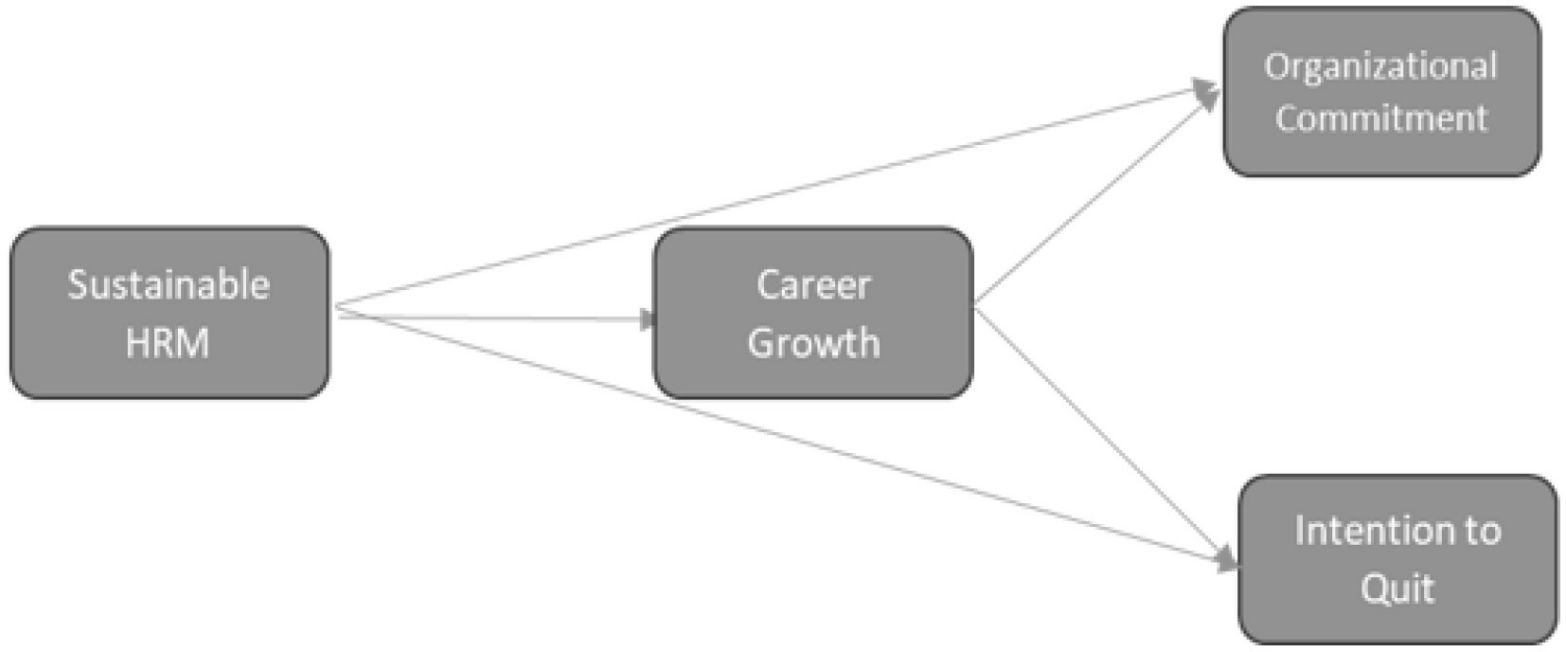
Research model.

The proposed hypotheses were tested using PLS-SEM in SmartPLS 4.0, in conjunction with a non-parametric bootstrapping procedure. Specifically, 5,000 bootstrap subsamples were generated by random sampling with replacement from the original dataset. Path coefficients were estimated for each subsample, and *t*-statistics were computed based on the stability of these coefficients across the bootstrap distribution to evaluate the statistical significance of the hypothesized relationships (see Figure 2). Table 4 summarizes the hypothesis-testing results, reporting standardized path coefficients (β), *t*-values, and *p*-values for each hypothesized path. As a robustness check, age and organizational tenure were also included as control variables predicting career growth, organizational commitment, and turnover intention.

**Table 4:** Hypothesis testing results (direct effects).

Hypothesis	Path	β	SE	*t*	*p*	Result
H1	Sustainable HRM → Career growth	0.895	0.013	67.428	<0.001	Supported
H2	Sustainable HRM → Organizational commitment	0.325	0.075	4.304	<0.001	Supported
H3	Sustainable HRM → Turnover intention (reverse-coded)	0.284	0.070	4.067	<0.001	Supported
H4	Career growth → Organizational commitment	0.603	0.074	8.167	<0.001	Supported
H5	Career growth → Turnover intention (reverse-coded)	0.619	0.071	8.723	<0.001	Supported
–	age → Career Growth	−0.018	0.025	0.714	0.475	–
–	age → Organizational Commitment	−0.031	0.025	1.278	0.202	–
–	age → intention to quit(reverse-coded)	−0.003	0.024	0.112	0.911	–
–	tenure → Career Growth	0.012	0.023	0.499	0.618	–
–	tenure → Organizational Commitment	−0,035	0,029	1.231	0.219	–
–	tenure → intention to quit(reverse-coded)	−0,036	0,028	1.295	0.196	–

Bootstrapped standard errors (SE) and t-values are reported.

Intention to quit is reverse-coded such that higher values indicate lower intention to quit; thus, positive coefficients involving this construct should be interpreted as reductions in intention to quit.

**Figure 3:**
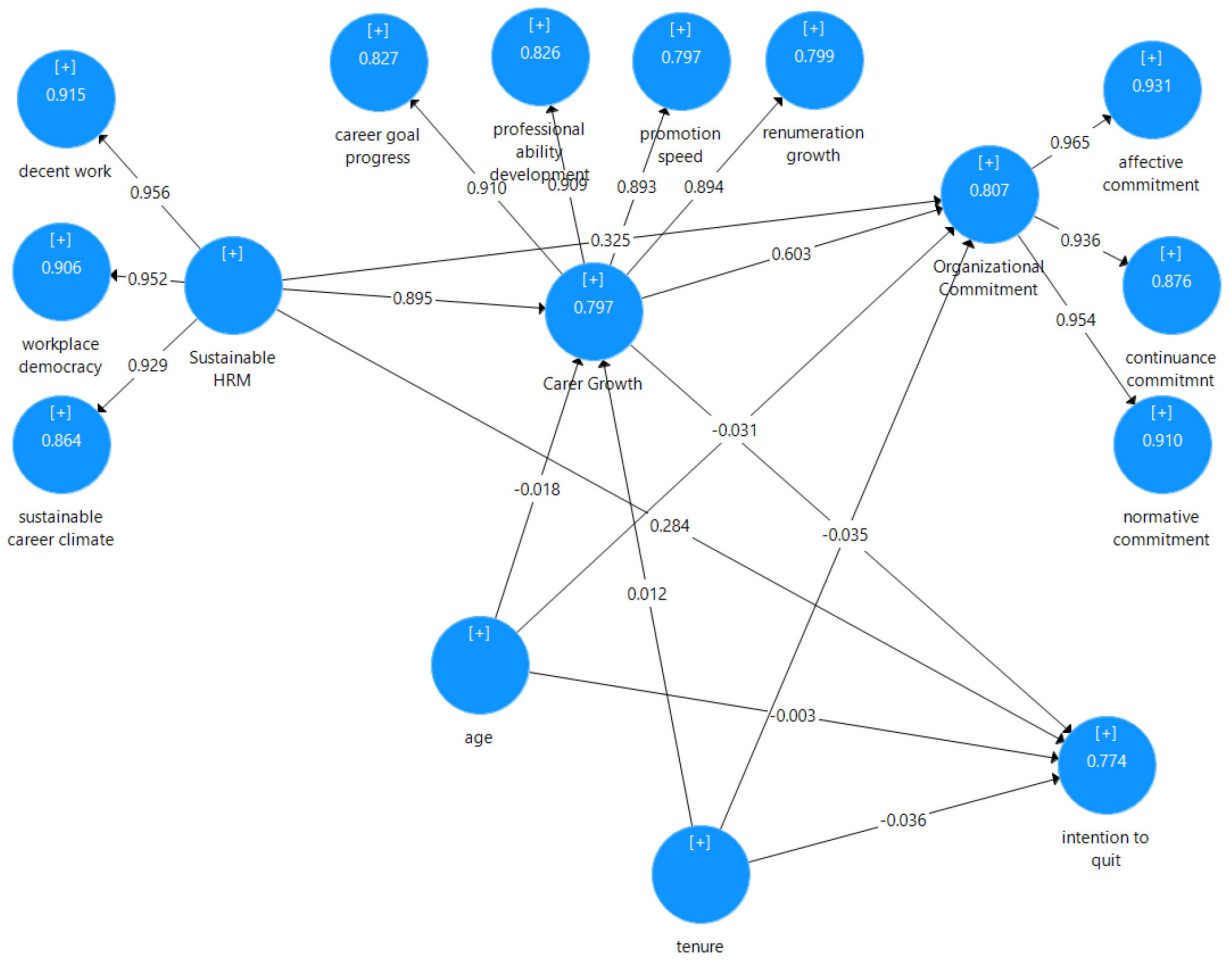
Structural model results (PLS-SEM): standardized path coefficients (β); *R*^2^ values shown in endogenous constructs.

Importantly, turnover intention was administered in fully reverse wording and coded such that higher scores indicate lower intention to quit. Accordingly, positive structural coefficients involving this construct should be interpreted as reductions in intention to quit (i.e., lower turnover intentions). The results (see Figure 3) indicate a very strong positive relationship between sustainable HRM practices and career growth (β = 0.895, *p* < 0.001), providing support for H1. This suggests that employees who perceive stronger sustainable HRM practices also report higher career growth. The path from sustainable HRM practices to organizational commitment is also positive and significant (β = 0.325, *p* < 0.001), supporting H2. In addition, sustainable HRM practices are positively related to reverse-coded turnover intention (β = 0.284, *p* < 0.001), supporting H3; given the coding direction, this finding indicates that sustainable HRM practices are associated with lower intention to quit. Career growth is positively related to organizational commitment (β = 0.603, *p* < 0.001), supporting H4, suggesting that perceived career advancement is closely associated with employees' attachment to the organization. Finally, career growth is positively related to reverse-coded turnover intention (β = 0.619, *p* < 0.001), supporting H5; consistent with the coding direction, this result indicates that higher perceived career growth is associated with lower intention to quit.

The robustness analysis further showed that neither age nor organizational tenure had statistically significant effects on career growth, organizational commitment, or reverse-coded turnover intention (all *p* > 0.05). Importantly, the inclusion of these control variables did not materially alter the direction or statistical significance of the focal structural relationships, suggesting that the substantive pattern of findings remained stable after accounting for demographic variation in age and tenure.

A mediation analysis was conducted to examine whether career growth transmits the effects of sustainable HRM practices to organizational commitment and intention to quit. The mediation results are reported in Table 5, which presents the total, direct, and specific indirect effects, along with variance accounted for (VAF) and bootstrapped confidence intervals.

**Table 5:** Mediation analysis (bootstrapped effects).

Hypothesis	Mediation path	Total effect β (*t*)	Direct effect β (*t*)	Indirect effect β (*t*)	Bootstrapped 95% CI (LL, UL)	VAF	Mediation type
H6	Sustainable HRM → Career growth → Organizational commitment	0.864 (48.113)	0.325 (4.304)	0.539 (8.136)	[0.414, 0.665]	0.624	Partial mediation
H7	Sustainable HRM → Career growth → Turnover intention (reverse-coded)	0.838 (42.883)	0.284 (4.067)	0.554 (8.733)	[0.425, 0.679]	0.661	Partial mediation

Turnover intention is reverse-coded such that higher values indicate lower intention to quit; therefore, positive coefficients imply reductions in intention to quit.

Bootstrapped confidence intervals are based on 5,000 resamples; indirect effects are considered significant when the confidence interval does not include zero.

Regarding organizational commitment, the total effect of sustainable HRM practices on organizational commitment was positive and significant (β = 0.864, *t* = 48.113, and *p* < 0.001). When career growth was included as a mediator, the direct effect decreased but remained significant (β = 0.325, *t* = 4.304, and *p* < 0.001), indicating partial mediation. Consistent with this interpretation, the specific indirect effect via career growth was also positive and significant (β = 0.539, *t* = 8.136, and *p* < 0.001). The VAF for this pathway was 62.4%, which falls within the conventional 20–80% range and therefore supports partial mediation. The robustness of this indirect effect is further supported by the bootstrapped 95% confidence interval for the indirect effect ([0.414, 0.665]), which does not include zero.

Turning to intention to quit, it is important to note that this construct was reverse-coded such that higher values indicate lower intention to quit. Accordingly, positive coefficients in paths involving this construct should be interpreted as associations with lower intention to quit. The total effect of sustainable HRM practices on reverse-coded turnover intention was positive and significant (β = 0.838, *t* = 42.883, and *p* < 0.001), indicating that higher sustainable HRM practices are associated with lower intention to quit. After introducing career growth as a mediator, the direct effect of sustainable HRM practices on reverse-coded turnover intention decreased but remained significant (β = 0.284, *t* = 4.067, and *p* < 0.001), again indicating partial mediation. The specific indirect effect via career growth was also positive and significant (β = 0.554, *t* = 8.733, and *p* < 0.001), showing that career growth transmits a substantial portion of the association between sustainable HRM practices and lower intention to quit. The VAF for this pathway was 66.1%, which also indicates partial mediation. The bootstrapped 95% confidence interval for the indirect effect ([0.425, 0.679]) does not include zero, confirming the robustness of the mediation effect.

Overall, these findings suggest that career growth functions as a meaningful mediating mechanism through which sustainable HRM practices are associated with stronger organizational commitment and lower intention to quit among employees.

### Structural model

Table 6 presents a detailed evaluation of the structural model's quality using the Partial Least Squares Structural Equation Modeling (PLS-SEM) approach. Key indicators, including the coefficient of determination (*R*^2^), predictive validity (*Q*^2^), and the standardized root mean squared residual (SRMR), were used to assess the model's fit and its predictive capacity for the dependent variables (see Table 6). The *R*^2^ values indicate the proportion of variance in the dependent constructs explained by the independent constructs. Following Chin's (2001) criteria, *R*^2^ values are classified as small (0.02–0.13), medium (0.13–0.26), or large (0.26 and above). In the present model, the *R*^2^ value for Career Growth is 0.797, indicating that the predictors explain a substantial proportion of the variance in career growth. Similarly, the *R*^2^ for Organizational Commitment is 0.807, suggesting strong explanatory power for organizational commitment. The *R*^2^ value for Turnover Intention is 0.774, likewise indicating that the model explains a substantial proportion of variance in this construct. Collectively, these values suggest that the structural model has considerable explanatory power across the three endogenous variables.

**Table 6:** Structural model explanatory and predictive accuracy.

Endogenous construct	*R* ^2^	*Q* ^2^
Career growth	0.797	0.595
Organizational commitment	0.807	0.630
Turnover intention	0.774	0.617

SRMR (model fit) = 0.056.

The *Q*^2^ values assess the predictive relevance of the model by determining how well it predicts data points for the dependent constructs. Positive *Q*^2^ values confirm that the model has predictive validity. In this study, the *Q*^2^ value for Career Growth is 0.595, the *Q*^2^ value for Organizational Commitment is 0.630, and the *Q*^2^ value for Turnover Intention is 0.617. These consistently positive values indicate that the model has substantial predictive relevance for all three endogenous constructs.

The SRMR value for the overall model is 0.056. According to Hu and Bentler's (1999) criteria, an SRMR value below 0.08 indicates acceptable model fit. This result suggests that the discrepancy between the observed correlations and the model-implied correlations is low, supporting the overall fit of the structural model. In conclusion, the analysis demonstrates that the structural model exhibits strong explanatory power, substantial predictive relevance, and acceptable overall fit. Taken together, the *R*^2^, *Q*^2^, and SRMR values indicate that the model performs well in accounting for variation in career growth, organizational commitment, and turnover intention.

## Discussion

This study examined whether sustainable HRM practices are associated with organizational commitment and intention to quit among Generation Z employees, and whether these relationships operate through perceived career growth. The findings suggest that sustainable HRM may be more consequential when employees experience it as a coherent and development-oriented system rather than as a set of isolated HR activities. In our model, sustainable HRM was positively related to perceived career growth; career growth, in turn, was positively associated with organizational commitment and negatively associated with intention to quit. This substantive pattern also remained stable when age and organizational tenure were included as control variables, suggesting that the reported associations are not primarily attributable to demographic overlap within the early-career Gen Z sample. In addition, the mediation results are consistent with the idea that career growth functions as one relevant pathway through which sustainable HRM is associated with these two retention-relevant outcomes.

These findings contribute to a more focused understanding of how sustainable HRM becomes psychologically consequential for younger employees. Prior discussions of sustainable HRM have often emphasized values, wellbeing, and long-term organizational responsibility (Aust et al., 2020; Leslie et al., 2021; Meret et al., 2018). Our results suggest that, for Generation Z employees, the importance of such systems may lie not only in their normative appeal but also in whether they are interpreted as creating meaningful prospects for future development. In other words, sustainable HRM appears to matter partly because it helps employees evaluate whether the organization offers a viable pathway for growth, continuity, and longer-term membership.

The role of career growth is especially notable. Rather than functioning as a peripheral developmental outcome, career growth appears to be a meaningful explanatory link between sustainable HRM and employee attachment. This is theoretically meaningful because it shows that younger employees do not respond only to the presence of HR practices *per se*; they also respond to whether these practices are experienced as creating skill development, advancement prospects, and a credible future within the organization. For Generation Z employees, who are often described as attentive to learning, progression, and meaningful work, these perceptions may be particularly relevant in shaping both stronger commitment and lower withdrawal cognitions (Dimock, 2019; Xueyun et al., 2023; Ayoobzadeh et al., 2024).

At the same time, the mediation observed in this study is partial rather than complete. This suggests that sustainable HRM may influence organizational commitment and intention to quit through additional pathways beyond career growth. Such a pattern is theoretically plausible. Employees may also respond to sustainable HRM through other mechanisms, such as perceived organizational support, person-organization fit, engagement, or procedural fairness. Accordingly, our findings do not imply that career growth is the only relevant process, but they do suggest that it may be one of the more relevant and managerially meaningful channels through which sustainable HRM is linked to retention-related outcomes among Generation Z employees.

A further implication of the findings is that sustainable HRM should be understood as a system-level signal rather than as a collection of disconnected practices. In this study, decent work, workplace democracy, and a career-supportive climate were modeled as an integrated higher-order construct. The pattern of results is consistent with the view that employees respond not merely to single HR practices in isolation, but also to the broader architecture those practices collectively create. This is useful because it points attention toward the internal consistency of the HRM system as experienced by employees. For younger workers in particular, such coherence may shape whether organizational promises are interpreted as meaningful, durable, and worth reciprocating.

### Theoretical contributions

This study offers three main theoretical contributions. First, it contributes to the sustainable HRM literature by clarifying a specific mechanism through which sustainable HRM is linked to retention-related employee outcomes. Rather than treating sustainable HRM as a broad value orientation with diffuse consequences, the present study specifies perceived career growth as a meaningful transmission pathway through which sustainable HRM relates to stronger organizational commitment and lower intention to quit. In doing so, the study moves the conversation from whether sustainable HRM matters to how it matters for early-career employees.

Second, the study speaks more directly to the connection between sustainable HRM and career-related perspectives by linking the model to Social Cognitive Career Theory and Psychological Contract Theory. From an SCCT perspective, sustainable HRM may shape employees' development-related beliefs by supporting learning, participation, and future-oriented progression opportunities. From a psychological contract perspective, such practices may also be interpreted as signals that the organization is willing to invest in employees beyond short-term exchange, thereby strengthening attachment and lowering intentions to leave. Taken together, these perspectives help explain why sustainable HRM may become especially influential when employees interpret it as supporting a meaningful future within the organization.

Third, the study contributes to research on Generation Z by showing that career growth may be more than a desirable job attribute for this cohort and may also function as an important evaluative lens through which employment relationships are interpreted. Much of the emerging literature portrays Generation Z employees as particularly attentive to development, flexibility, values, and future opportunity (Maloni et al., 2019; Schroth, 2019; Xueyun et al., 2023). Our findings refine this literature by showing that sustainable HRM is associated with stronger attachment and lower quitting intention partly because it is linked to perceptions of career growth. This extends existing work by positioning growth perceptions not simply as an outcome of HR practices, but as a mechanism that helps explain why those practices are consequential for younger employees.

Finally, the study offers a more parsimonious conceptual contribution than earlier drafts of the manuscript implied. Rather than advancing a new unmeasured explanatory construct, the present contribution lies in aligning the theorized and tested model more closely around sustainable HRM, perceived career growth, organizational commitment, and intention to quit. Future research may examine whether variables such as process visibility or perceived transparency further shape these relationships, but the present study's theoretical value lies in showing that career growth provides a meaningful explanatory pathway within the tested model.

### Managerial implications

The findings suggest that organizations seeking to strengthen commitment and reduce quitting intention among Generation Z employees should focus not only on offering supportive HR practices, but also on ensuring that these practices are experienced as developmentally meaningful. Sustainable HRM appears to be more effective when employees can connect organizational policies to their own future prospects. Accordingly, organizations may benefit from designing HR systems that make learning, advancement, and longer-term progression tangible parts of everyday employment rather than symbolic promises.

One implication is that career development should be treated as a core retention practice rather than a secondary HR benefit. Managers may benefit from building clearer developmental pathways through role ladders, competency maps, structured feedback routines, internal mobility options, and regular career conversations. Such practices may help employees understand how effort, skill development, and organizational participation translate into future opportunity. For Generation Z employees in particular, this kind of developmental clarity may strengthen the sense that staying in the organization is both worthwhile and sustainable.

The findings also suggest that sustainable HRM should be enacted consistently across multiple domains of the employment relationship. Decent work conditions, participative arrangements, and a career-supportive climate may be less effective when managed as separate agendas. When these elements are aligned, employees are more likely to interpret the organization as supportive, credible, and future-oriented. This implies that retention strategies for younger employees should be built around coherent systems rather than fragmented interventions. For example, training opportunities may have limited impact if they are not accompanied by fair access, visible developmental use, and follow-up discussions about progression.

A further practical implication is that organizations should monitor developmental outcomes, not only turnover outcomes. Waiting until employees are already considering leaving may limit the organization's ability to respond proactively. Instead, organizations should track earlier indicators such as perceived career growth, access to development, internal mobility rates, participation in mentoring or skill-building programs, and employee perceptions of future opportunity. These indicators may provide a more immediate signal of whether sustainable HRM is translating into stronger attachment among Generation Z employees.

In this sense, one practical implication is that organizations should be cautious about assuming that sustainability-oriented HR messages will automatically translate into loyalty. Employees are more likely to remain committed when those messages are supported by concrete developmental experiences. Sustainable HRM is therefore likely to be most effective when it creates not only favorable conditions of work, but also a credible basis for younger employees to envision growth and continuity within the organization.

### Limitations and future research

This study has several limitations that should be acknowledged. First, the research is based on a cross-sectional design, which limits causal inference. Although the hypothesized relationships are theory-driven, the observed associations should not be interpreted as definitive evidence of causality. Future studies should adopt longitudinal, panel, or time-lagged designs to examine whether sustainable HRM predicts later changes in career growth, organizational commitment, and intention to quit over time. Such designs would also help address the possibility of reciprocal relationships.

Second, the data rely on self-reports collected from a single source. Although procedural safeguards such as anonymity and neutral wording were used, common method bias and social desirability bias cannot be fully ruled out. Future research could strengthen inference by using multi-source data, temporal separation, or administrative indicators such as internal mobility, promotion history, or actual turnover. Incorporating explicit checks for social desirability would also improve methodological confidence.

Third, the study focuses on Generation Z employees in a single national context and draws primarily on respondents in the younger segment of this cohort. This provides a theoretically coherent boundary condition, but it also limits generalizability. Because the sample is concentrated in a relatively narrow age range, age-related variation is intentionally limited, and age and organizational tenure may not be fully separable in this dataset. Although supplementary robustness checks including age and organizational tenure as control variables did not materially alter the substantive pattern of findings, the results should still be interpreted as relationships observed within a relatively homogeneous early-career Generation Z sample rather than as evidence that age plays no role in shaping these outcomes. Future research should compare narrower sub-groups within Generation Z, examine adjacent cohorts such as late Millennials, and test whether the observed relationships differ across sectors, occupations, and institutional settings. Such comparative designs would help clarify whether the role of career growth is equally salient across workforce segments and would allow age and tenure to be modeled more explicitly.

Fourth, the model was intentionally specified in a parsimonious manner, centering on sustainable HRM, career growth, organizational commitment, and intention to quit. While this focus enhances conceptual clarity, it also means that other potentially relevant explanatory variables were not included. Future studies should examine adjacent mechanisms and boundary conditions, such as perceived organizational support, engagement, workload, leadership style, labor-market opportunity, and person-organization fit. Testing such extensions would help determine whether career growth remains the most influential pathway or one of several parallel mechanisms.

Finally, future research may build on the present model by examining how employees interpret the implementation of career-related HR processes. Although the present study does not directly measure constructs such as process visibility or perceived transparency, these may help explain variation in how employees translate sustainable HRM into growth perceptions. Such variables should be explicitly operationalized and tested in future models rather than treated as background assumptions. This would allow scholars to examine more directly whether they mediate, moderate, or simply contextualize the sustainable HRM–career growth–retention pathway.

## Data Availability

The raw data supporting the conclusions of this article will be made available by the authors, without undue reservation.
