# Supplementary material for: Sustainable HR practices and Generation Z: role of career growth in commitment and turnover intention
Source: Front Psychol. 2026 May 18;17:1819084. doi: 10.3389/fpsyg.2026.1819084 (PMC13222802; doi:10.3389/fpsyg.2026.1819084)
Supplement: Supplementary file 1 [file Supplementary_file_1.pdf]

APENDIX

Appendix Measure

Standardized loadings are in parentheses.

* denotes the dropped item; either they reduce the AVE to less than .50, or they have low loading weights.

SUSTAINABLE HRM PRACTICES adapted from de Prins et al.(2020)

Decent Work

Jobs are a reflection of what employees are good at and like to do in our organization. (0.781)

Employees are largely able to manage their work themselves. (0.845)

This organization truly cares about the well-being of employees. (0.808)

Employees are rewarded fairly and equitably according to the effort they put into their work. (0.852)

Workplace Democracy

The organization has no unnecessary hierarchical levels. (0.804)

The decision-making in this organization is highly centralized (R). (0.840)

Bottom-up voice is stimulated in the organization. (0.830)

Managers appreciate and anticipate the suggestions and ideas of employees. (0.854)

Sustainable Career Climate

There is a range of training and learning opportunities for everyone in the organization. (0.891)

The organization helps employees to maintain long-term employability and agility. (0.862)

Employees receive regular feedback on their performance and results. (0.883)

CAREER GROWTH adapted from Weng et al. (2010).

Career Goal Progress

My present job moves me closer to my career goals. (0.870)

My present job is relevant to my career goals and vocational growth. (0.874)

My present job sets the foundation for the realization of my career goals. (0.890)

My present job provides me with good opportunities to realize my career goals. (0.875)

Professional Ability Development

My present job encourages me to continuously gain new and job-related skills. (0.890)

My present job encourages me to continuously gain new job-related knowledge. (0.855)

My present job encourages me to accumulate richer work experiences. (0.859)

My present job enables me to continuously improve my professional capabilities. (0.894)

Promotion Speed

My promotion speed in the present organization is fast. (0.814)

The probability of being promoted in my present organization is high. (0.800)

Compared with previous organizations, my position in my present one is ideal. (0.793)

Compared with my colleagues, I am being promoted faster. (0.847)

Remuneration Growth

My salary is growing quickly in my present organization. (0.907)

In this organization, the possibility of my current salary being increased is very large. (0.832)

Compared with my colleagues, my salary has grown more quickly. (0.924)

ORGANIZATIONAL COMMITMENT adapted from Allen and Meyer (1996)

Affective Commitment

I would be very happy to spend the rest of my career in this organization. (0.779)

I really feel as if this organization's problems are my own. (0.804)

I do not feel like 'part of my family' at this organization. (0.888)

I do not feel 'emotionally attached' to this organization. (0.875)

This organization has a great deal of personal meaning for me. (0.884)

I do not feel a strong sense of belonging to this organization. (0.846)

Continuance Commitment

It would be very hard for me to leave my job at this organization right now even if I wanted to.(0.719)

Too much of my life would be disrupted if I leave my organization.*

Right now, staying with my job at this organization is a matter of necessity as much as desire. *

I believe I have too few options to consider leaving this organization. (0.791)

One of the few negative consequences of leaving my job at this organization would be the scarcity of available alternatives elsewhere. (0.839)

One of the major reasons I continue to work for this organization is that leaving would require considerable personal sacrifice. (0.709)

Normative Commitment

I do not feel any obligation to remain with my organization (R). *

Even if it were to my advantage, I do not feel it would be right to leave. (0.781)

I would feel guilty if I left this organization now. (0.790)

This organization deserves my loyalty. (0.790)

I would not leave my organization right now because of my sense of obligation to it. (0.775)

I owe a great deal to this organization. (0.861)

INTENTION TO QUIT adapted from Erkmen and Çam Kahraman (2021), which is originally developed by Bothma and Roodt (2013)

I look forward to another day at work (R) (0.776)

While doing my job, I never imagine receiving a job offer from another organization. (R) (0.757)

Recently, I have not experienced a persistent desire to leave my current workplace (R). (0.797)

I can think of compelling reasons to remain with this company for many more years. (R) (0.778)

I never consider leaving my job. (R) (0.760)

I doubt I could find another workplace comparable to my current one (R). (0.718)

The prospect of being forced to leave this company would deeply upset me (R). (0.767)

After completing the task I am currently responsible for, I would prefer to stay with this company rather than leave. (R) (0.727)
